# 보조생식술을 받는 여성의 불확실성과 배우자 지지가 난임 관련 삶의 질에 미치는 영향

**DOI:** 10.4069/kjwhn.2020.03.15

**Published:** 2020-03-25

**Authors:** Hye Shin Lee, Sunjoo Boo, Jeong-Ah Ahn, Ju-Eun Song

**Affiliations:** College of Nursing, Ajou University, Suwon, Korea; 아주대학교 간호대학; College of Nursing·Research Institute of Nursing Science, Ajou University, Suwon, Korea; 아주대학교 간호대학·간호과학연구소

**Keywords:** Infertility, Assisted reproductive technologies, Quality of life, Uncertainty, Spouses, 난임, 보조생식술, 삶의 질, 불확실성, 배우자 지지

## Abstract

**Purpose:**

The purpose of this study was to investigate the effects of uncertainty and spousal support on infertility-related quality of life (QoL) in women undergoing assisted reproductive technologies.

**Methods:**

In this correlational survey study, 172 infertile women undergoing assisted reproductive technologies for infertility treatment at M hospital in Seoul participated. Data collection took place at the outpatient department of M hospital using a self-report questionnaire from July to August 2019. Data were analyzed using SPSS for Windows version 28.0.

**Results:**

The mean scores for uncertainty, spousal support, and infertility-related quality of life (QoL) were 28.35 (out of 50), 86.67 (out of 115), and 57.98 (out of 100), respectively. Infertility-related quality of life (QoL) was positively correlated with spousal support and negatively correlated with uncertainty. According to the regression analysis, infertility-related quality of life (QoL) was significantly affected by uncertainty, total number of assisted reproductive technology treatments, marriage duration, subjective health status, the financial burden of infertility testing, and the presence of a burdensome person. These variables had an explanatory power of 35.0% for infertility-related quality of life (QoL).

**Conclusion:**

Uncertainty was an important factor influencing infertility-related quality of life (QoL) among women undergoing assisted reproductive technologies. It is necessary to develop and implement a nursing intervention program focused on reducing various forms of uncertainty during assisted reproductive procedures and to consider other factors affecting infertility-related quality of life (QoL) in the clinical setting.

## Introduction

### 연구 필요성

최근 한국은 초저출산이 중요한 사회적 이슈가 되고 있으며, 이의 극복을 위한 방안의 하나로서 증가하는 난임 인구에 대한 지원이 중요한 국가정책의 하나로 떠오르고 있다. 실제로 한국의 난임 진단자 수는 2014년부터 20만 명을 넘어 매년 증가 추세이며, 2016년 22만 1,261명으로 지속적으로 증가하고 있다[1]. 이에 대한 국가적 지원 정책으로 2006년부터 난임 부부 지원사업이 시작됨에 따라 해마다 난임 시술 건수도 증가하고 있다. 구체적으로 2016년 기준으로 300개 이상의 난임 센터에서 연간 8만 건 이상의 보조생식술이 시술되고 있으며, 체외수정 건수는 2014년 40,555건에서 2016년 52,689건으로 증가하였고, 체외수정으로 출생한 신생아 수 역시 2014년 1만 1,597명에서 2016년 1만 4,373명으로 증가하고 있다[1].

난임은 여성들에게 중요한 삶의 목표를 조정해야 하는 만성적이고 도전적인 건강 문제이기 때문에[2], 흔히 난임의 진단과 시술을 받는 절차 그 자체가 삶의 질을 저하하는 요인이 된다. 특히 보조생식술로서 설명되는 다양한 난임 치료를 받는 과정에서 난임 여성들은 신체적 스트레스뿐 아니라 불안과 우울, 조급함 및 상실감 등의 다양한 정신적 고통으로 사회 생활에서의 위축을 겪는다[3]. 이러한 모든 난임의 진단과 치료과정은 난임 여성의 심리적 스트레스를 증가시켜 삶의 질에 부정적인 영향을 가져올 수 있다[2]. 난임 여성의 삶의 질에 영향을 미치는 요인으로 국내 선행연구에서는 우울, 스트레스 치료비용에 대한 부담감 등 다양한 요인이 탐색되었다[4-6]. 이는 난임 여성 개인적 차원의 부정적 요소가 삶의 질에 부정적인 영향을 주는 것으로, 이러한 부정적인 요소들을 해결하여 난임 여성들의 삶의 질을 높이려는 노력은 매우 중요할 수 있으며, 중재 가능한 관련 요인들을 지속적으로 탐색하고 이를 임상 현장에 반영하기 위한 노력이 필요하다고 본다.

여러 부정적인 요소 중 난임 여성이 가장 흔히 경험하게 되는 불확실성[7]은 임신 성공까지의 기간이 얼마나 될지 모르는 것으로, 난임 여성은 이로 인해 불안과 답답함이 가중되고 나아가 다양한 부정적 정서를 경험하게 된다[7]. 흔히 인공수정의 임신 성공률은 10–20%, 체외수정의 임신 성공률은 30–40%로 보고되는 것을 고려할 때[3], 난임 시술을 받는 여성들은 임신 성공에 대한 불확실성뿐 아니라 배우자와의 관계에 있어서의 불확실성 등 다양한 불확실성을 경험하는 것으로 설명하고 있다[7,8]. 이러한 불확실성은 체외수정 단계를 거치면서 더욱더 가중되므로[8] 삶의 질을 떨어뜨리는 데 주요한 요인이 될 수 있으나, 난임 여성에서 불확실성이 삶의 질에 미치는 직접적인 영향을 규명한 연구는 드물다. 불확실성은 유방암 환자의 삶의 질을 직접적으로 낮추는 요인으로 설명된 바 있으며[9], 난임 여성보다는 타 질환군에서 직접적인 관련성이 설명되었다. 따라서 난임 여성이 가장 흔히 경험하는 것으로 보고되는 불확실성이[7] 삶의 질을 유의하게 낮추는 영향요인인지를 규명할 필요가 있다.

또한, 배우자 지지는 여러 선행논문에서 직·간접적인 삶의 질 영향요인으로 설명되고 있다[5,6,10-12]. 배우자 지지는 난임 치료과정에 있는 난임 여성의 삶의 질을 높이는 직접적인 영향요인인 동시에[5,6], 배우자 지지가 높은 난임 여성들이 배우자와 함께 아이를 가지기 위해 노력하는 과정에서 부부 친밀감을 확인하고 이러한 요인이 결혼생활 만족도에도 긍정적인 영향을 줌으로써[10] 난임 여성의 삶의 질에 간접적으로 영향을 주는 요인이 될 수 있다[5,6,11]. 반면에 난임 진단 이후 배우자와의 관계가 나빠졌다고 인식하게 되는 경우 매우 심각한 수준의 우울 증상[12] 및 부부의 의사소통과 관계의 변화도 유발될 수 있어 난임을 극복하기 어렵게 만들 수 있고, 이는 나아가 난임 여성의 삶의 질을 낮추는 요인이 될 수 있다[5,6]. 난임을 극복하는 과정은 개인의 문제만이 아니고 부부가 함께 해결해야 할 공동 과업으로 접근하여야 하므로, 현대 사회에서 가장 중요한 지지 체계인 배우자 지지 정도가 난임 관련 삶의 질에 미치는 영향을 재확인하는 것이 필요하다.

이제껏 난임 여성의 삶의 질에 대한 국내, 외 선행연구들을 살펴보면 조사연구가 주를 이루는데, 삶의 질의 영향요인으로 우울, 스트레스, 치료비용 부담감, 부부 적응, 가족 지지, 극복력 등이 난임 여성의 삶의 질에 영향을 미치는 것으로 나타났으며[4-6,11], 보조생식술 여성을 대상으로 제공한 상담 같은 심리적 중재가 임신율에 미치는 효과에 대한 메타분석 연구와[13] 삶의 질에 대한 체계적 문헌고찰 연구[14] 등이 진행된 바 있다. 하지만, 국내에서는 난임 여성이 경험하는 난임 관련 삶의 질에 관한 연구가 아직 부족하여, 난임 대상자를 위한 임상 중재 프로그램을 계획하는 데 필요한 기초자료가 충분치 못한 실정이다. 아울러 보조생식술을 받는 여성은 치료에 따른 복잡한 감정들을 경험하고 시술 과정 동안 많은 스트레스에 노출되고 있어[8] 이들만을 대상으로 연구를 진행할 필요가 있으나, 보조생식술을 받는 난임 여성만을 대상으로 한 연구는 드물다.

이에 본 연구에서는 보조생식술을 받는 난임 여성들의 내적 특성인 불확실성 정도와 외적 자원인 배우자 지지 정도를 파악하고 난임 관련 삶의 질에 영향을 미치는 정도를 규명하고자 하며, 이를 근간으로 난임 여성의 삶의 질을 향상할 수 있는 간호 중재 개발의 방향을 제시하고자 한다.

### 연구 목적

본 연구의 목적은 불확실성과 배우자 지지가 보조생식술을 받는 난임 여성의 삶의 질에 미치는 영향을 파악하기 위함이며, 구체적인 목적은 다음과 같다.

1) 대상자의 불확실성, 배우자 지지 및 난임 관련 삶의 질의 정도를 파악한다.

2) 대상자의 불확실성, 배우자 지지 및 난임 관련 삶의 질 간의 상관관계를 파악한다.

3) 대상자의 일반적 특성과 난임 관련 특성에 따른 난임 관련 삶의 질 차이를 파악한다.

4) 대상자의 불확실성과 배우자 지지가 난임 관련 삶의 질에 미치는 영향을 파악한다.

## Methods

Ethics statement: This study was approved by the Institutional Review Board of Ajou University Hospital (No. AJIRB-MEDSUR-19-191). Informed consent was obtained from the subjects.

### 연구 설계

본 연구는 불확실성과 배우자 지지가 보조생식술을 받는 난임 여성의 삶의 질에 미치는 영향을 규명하기 위한 상관성 조사연구이다.

### 연구 대상

본 연구의 대상자는 서울에 소재한 난임 전문병원인 M 산부인과에 진료를 위해 방문한 여성들 중에서 원발성 또는 속발성 난임으로 진단받고, 현재 체외수정의 보조생식술을 1회 이상 받은 난임 여성이며, 연구의 목적을 이해하고 자발적 참여에 동의한 난임 여성 190명을 편의표집하여 자료수집을 하였다. 이 중 연구 참여 중도 포기 및 작성 오류로 인한 설문지 18부를 제외하여 총 172명이 연구대상에 포함되었다. 본 연구에서 보조생식술을 받는 난임 여성을 대상자로 선정한 이유는 선행연구에서 난임 시술을 받지 않는 난임 여성보다 보조생식술을 받는 난임 여성이 시술 과정 동안 신체적 고통과 더불어 상대적으로 스트레스, 우울, 조급함, 고독감 등의 다양한 정서적 반응과 정신적 고통을 경험하는 것으로 나타났기 때문으로[3], 보조생식술 과정에 있는 난임 여성을 위한 보다 적절한 삶의 질 향상 중재 프로그램 개발의 기초자료를 제공하기 위함이다.

본 연구에서는 주요 통계기법으로 위계적 다중회귀분석(hierarchical multiple regression analysis)을 이용하였으며, 난임 관련 삶의 질 관련 요인으로 불확실성과 배우자 지지의 주요 독립변수 외에, 단변량 분석을 통해서 난임 관련 삶의 질 정도에 통계적으로 유의한 차이를 보였던 나이, 결혼 기간[11], 주관적 건강 상태[11], 보조생식술 횟수[12], 배란유도 방법[15], 난임 검사비용 부담 여부[5,10,11], 난임에 부담주는 사람 유무[16] 등이 함께 회귀분석에 포함되었다. G*Power 3.1 프리웨어를 이용하여 유의수준 .05, 선행연구에서 사용된 중간효과 크기 .15 [5,6,10,11], 검정력 .80, 예측변수의 수 9개일 때 필요한 최소 표본수는 114명이었으므로, 본 연구의 표본수 172명은 분석을 위한 최소 표본수를 충족하였다.

### 연구 도구

#### 일반적 특성과 난임 관련 특성

일반적 특성과 난임 관련 특성은 연구자가 선행연구[3-6,11]를 근거로 하여 선정하였으며, 나이, 결혼 기간, 주관적 건강 상태, 교육 정도, 직업, 경제 상태, 유산 경험, 현재 아이를 가장 바라는 사람, 난임 원인, 보조생식술 총 횟수, 배란유도 방법, 이식 방법, 난임 치료기간, 난임 검사 및 시술비용, 난임 상담 필요성, 난임에 부담 주는 사람 유무 등 총 19문항으로 구성하였다.

#### 불확실성

불확실성이란 질병의 치료과정과 관련된 상황과 자극에 대하여 정확히 알지 못하거나 모호하게 받아들이는 것을 의미한다[17]. 본 연구에서 불확실성 측정도구는 Kim과 Kim [17]이 불임 여성의 불확실성을 측정하기 위해 개발한 도구를 사용하였으며, 도구 사용에 대한 저자의 허락을 받았다. 이 도구는 불임 여성이 경험하는 관계적 측면의 불확실성에 관한 4개 문항과 개인적 측면의 불확실성 6개 문항의 총 10개 문항으로 구성되어 있으며, 각 문항은 ‘매우 확신한다(1점)’에서 ‘전혀 확신할 수 없다(5점)’의 Likert 5점 척도로 측정하여서 10점에서 50점의 점수 범위를 가지며, 점수가 높을수록 난임에 대한 불확실성 정도가 높음을 의미한다. 개발 당시 도구의 신뢰도 Cronbach’s α=.78이었고[17], 본 연구에서의 신뢰도는 Cronbach’s α=.89였다.

#### 배우자 지지

배우자 지지란 가족 내에서 부부간의 친밀한 상호작용으로 배우자에게 기대하는 사랑, 돌봄, 존중, 신뢰, 지지를 교환하는 것을 의미한다[18]. 본 연구에서 배우자 지지 측정도구는 Nam [18]이 개발한 도구를 Park [19]이 난임 여성에게 맞게 수정·보완한 도구를 사용하였으며, 도구 사용에 대한 저자의 허락을 받았다. 이 측정도구는 배우자의 사랑, 존중, 신뢰의 지지에 대한 9문항, 충분한 대화를 통한 지지에 대한 2문항, 실제적인 도움과 보살핌의 지지에 대한 12문항의 총 23개 문항으로 구성되어 있으며, 각 문항은 ‘전혀 그렇지 않다(1점)’에서 ‘항상 그렇다(5점)’의 Likert 5점 척도로 측정하여 23점부터 115점까지의 점수 범위를 가지며, 점수가 높을수록 배우자 지지 정도가 높음을 의미한다. 선행연구에서 도구의 신뢰도 Cronbach’s α=.95 였고[19], 본 연구에서의 신뢰도는 Cronbach’s α=.93이었다.

#### 난임 관련 삶의 질

삶의 질이란 개인이 사는 문화와 가치 체계에서 개인의 목표, 기대, 기준, 관심 등과 관련하여 개인이 인지하는 주관적인 안녕 상태를 말한다[20]. 본 연구에서 난임 관련 삶의 질 측정도구는 유럽생식배아협회(European Society of Human Reproduction & Embryology)와 미국생식의학회 (American Society of Reproductive Medicine)가 협력하여 개발한 국가별 표준화된 도구 중 한국어판 난임 관련 삶의 질 도구(Fertility Quality of Life [FertiQoL] tool)를 사용하였다[21]. 이 도구는 Cardiff 대학의 난임 관련 삶의 질 사이트에 다양한 언어로 공개되어 있어서 누구나 사용할 수 있는 도구이다. 전체적 건강 상태와 일반적인 삶의 질에 대한 2문항과, 난임 문제에 관한 정서영역 6개 문항, 심신영역 6개 문항, 관계영역 6개 문항, 사회영역 6개 문항의 총 24문항, 그리고 난임 치료에 관한 치료환경 6문항, 인내심 4문항의 10문항까지 전체 36개 문항으로 구성되어 있다. 건강 상태와 일반적인 삶의 질에 대한 2문항은 배경 정보로만 사용하며, 각 문항에 대해 0점에서 4점까지 5점의 Likert 척도로 측정하게 되어 있다. 총점은 0점에서 100점으로 환산한 점수로 평가하며, 총점 계산을 위해 총 영역과 하위 영역을 원시점수로 계산하고, 25에 각 영역의 관련 문항 수를 나눈 값에 각 영역별 점수를 곱한 값으로 산출한다. 점수가 높을수록 난임 관련 삶의 질 정도가 높은 것을 의미한다. 개발 당시 도구의 신뢰도 Cronbach’s α=.92였고[21], 본 연구에서 신뢰도는 Cronbach’s α=.93이었다.

### 자료 수집

자료수집은 2019년 7월 1일부터 2019년 8월 30일까지 서울에 소재한 M 난임 전문병원 외래에서 자가보고형 설문지를 통하여 진행되었다. 자료수집을 위해 먼저 난임 전문병원의 병원장과 간호부에 사전 협조를 구하였으며, 진료 대기 중인 연구대상자들에게 연구자가 직접 연구의 목적을 설명하고, 연구 참여에 동의할 시 직접 설문지를 배부하여 작성하게 한 후 그 자리에서 바로 회수하였다. 총 190명의 대상자에게 설문지를 배포하고, 총 172부를 회수하였다. 작성시간은 10–15분 정도 소요되었다. 설문 조사가 완료된 후에는 감사의 의미로 소정의 상품을 제공하였다.

### 자료 분석

수집된 자료는 IBM SPSS ver. 25.0 program (IBM Corp., Armonk, NY, USA)을 이용하여 분석하였다.

1) 대상자의 일반적 특성과 난임 관련 특성을 파악하기 위해 빈도, 평균, 표준편차, 백분율 등의 기술통계로 분석하였다.

2) 대상자의 불확실성, 배우자 지지, 난임 관련 삶의 질 정도는 평균과 표준편차로 분석하였다.

3) 대상자의 불확실성, 배우자 지지, 난임 관련 삶의 질 간의 상관관계는 Pearson correlation으로 분석하였다.

4) 대상자의 일반적 특성과 난임 관련 특성에 따른 난임 관련 삶의 질의 차이는 independent sample t-test와 one way ANOVA로 분석하였으며, 사후 검증은 Scheffé’s test로 검증하였다.

5) 대상자의 난임 관련 삶의 질 영향요인을 규명하기 위하여 위계적 회귀분석을 실시하였다.

## Results

### 연구대상자의 일반적 특성 및 난임 관련 특성

먼저 연구대상자의 일반적 특성을 살펴보면, 연구대상자의 평균 나이는 37.37세였으며, 결혼 기간은 평균 약 5.7년이었다. 주관적 건강 상태는 ‘건강하다’고 응답한 경우가 155명(90.1%)이었고, 교육 정도는 대학교 졸업이 143명(83.1%)으로 가장 많았으며, 직업은 ‘있다’가 110명(64.0%)으로 ‘없다’ 62명(36.0%)보다 많았다. 경제 상태는 ‘중’이 126명(73.3%)이었고, 종교는 무교가 69명(40.1%)으로 가장 많았다. 유산 경험은 ‘없음’이 107명(62.2%)이었으며, 현재 아이를 가장 바라는 사람으로는 남편이 102명(59.3%)으로 많았다.

또한 연구대상자의 난임 관련 특성을 살펴보면, 난임 원인은 원인불명인 경우가 83명(48.3%)으로 가장 많았고, 보조생식술 평균 횟수는 1.85회였다. 체외수정의 배란유도 방법은 과배란 유도방법이 124명(72.1%)으로 가장 많았다. 난임 치료기간은 평균 1.19년으로 나타났다. 난임 검사비용 부담감에 대한 응답으로는 ‘부담된다’가 134명(77.9%)으로 가장 많았다.

### 연구대상자의 불확실성, 배우자 지지 및 난임 관련 삶의 질 정도

연구대상자의 불확실성 정도는 평균 28.35±7.18점(문항 평균 2.84±0.72점)이었고, 관계적 측면의 불확실성은 평균 11.91±3.42점(문항 평균 2.90±0.86점)이었으며, 개인적 측면의 불확실성은 평균 16.43±4.84점(문항 평균 2.74±0.81점)이었다. 배우자 지지 정도는 평균 86.67±14.62점(문항 평균 3.77±0.64점)이었으며, 하위 요인인 사랑, 존중, 신뢰의 지지는 평균 33.36±5.87점(문항 평균 3.71±0.65점)이었고, 대화를 통한 지지는 평균 7.47±1.65점(문항 평균 3.73±0.82점), 도움과 보살핌의 지지는 평균 45.85±8.07점(문항 평균 3.82±0.67점)이었다. 난임 관련 삶의 질 정도는 100점 만점에 평균 57.98±12.96점(문항 평균 2.32±0.52점)이었다. 난임 치료에 관한 치료환경은 평균 52.74±12.77점(문항 평균 2.11±0.51점), 인내심은 평균 60.00±15.57점(문항 평균 2.40±0.62점)이었다(Table 1).

### 연구대상자의 불확실성, 배우자 지지 및 난임 관련 삶의 질의 상관관계

난임 관련 삶의 질은 불확실성과 유의한 음의 상관관계(r=–.28, *p*<.001)가 있었고, 배우자 지지와는 유의한 양의 상관관계(r=.23, *p*<.003)가 있었다(Table 2).

### 연구대상자의 특성에 따른 난임 관련 삶의 질 차이

연구대상자의 일반적 특성에 따른 난임 관련 삶의 질 차이를 분석한 결과 나이(t=–2.15, *p*=.035), 결혼 기간(t=4.32, *p*<.001), 주관적 건강 상태(t=2.28, *p*=.035)에서 통계적으로 유의한 차이가 나타났다(Table 3). 연구대상자의 난임 관련 특성에 따른 난임 관련 삶의 질 차이를 분석한 결과 보조생식술 총 횟수(F=13.51, *p*<.001), 배란유도 방법(F=8.08, *p*<.001), 난임 검사비용 부담감(F=4.94, *p*=.008), 난임에 부담 주는 사람(F=6.42, *p*<.001)에서 통계적으로 유의한 차이가 나타났다(Table 4).

### 연구대상자의 난임 관련 삶의 질에 미치는 영향요인

연구대상자의 난임 관련 삶의 질에 미치는 영향요인을 파악하기 위하여 위계적 다중회귀분석을 실시한 결과는 Table 5와 같다.

회귀모형을 검증하기 전 다중공선성(multicolinearity) 진단 결과 공차 한계(tolerance)의 범위가 0.51–0.99로 0.1 이상이었고, 분산 팽창인자(variance inflation factor, VIF)는 1.01–1.19로 기준치인 10을 넘지 않았으므로 다중공선성에 문제는 없었다. 또한 잔차분석 결과, Dubin-Watson 통계량이 1.706으로 2에 가까워 모형의 오차항 간에 자기 상관성 문제는 없는 것으로 나타났으며, Cook’s distance 값의 최댓값이 0.08로 1.0을 초과한 값이 없어 특이 값도 없는 것으로 나타났다.

분석 결과, 주요 독립변수의 효과를 분석한 모형 1에서는 불확실성(β=–.13, *p*=.001)이 높을수록, 배우자 지지(β=.07, *p*=.022)가 낮을수록 난임 관련 삶의 질은 낮은 것으로 나타났다. 본 회귀모형은 통계적으로 유의하였으며(F=10.15, *p*<.001), 이들 2개의 독립변수에 의한 난임 관련 삶의 질의 설명력은 10.7%로 나타났다. 모형 2에서는 단변량 분석에서 난임 관련 삶의 질과의 관련성이 있는 것으로 나타난 연구대상자의 일반적 특성과 난임 관련 특성들을 주요 독립변수와 함께 투여하여 회귀분석을 실시하였다. 분석 결과, 난임 관련 삶의 질에 영향을 미치는 요인으로 불확실성이 높을수록(β=–.19, *p*=.007), 보조생식술 총 횟수가 많을수록(β=–.24, *p*=.009), 결혼 기간이 증가할수록(β=–.16, *p*=.023) 난임 관련 삶의 질이 낮은 것으로 설명되었다. 또한 주관적 건강 상태가 안 좋다고 응답한 군(β=–0.22, *p*=.001), 난임 검사비용 부담이 있다고 응답한 군과(β=–.18, *p*=.007), 난임에 부담 주는 사람이 있다고 응답한 군(β=–.13, *p*=.047)에서 난임 관련 삶의 질이 낮은 것으로 설명되었다. 이들 변수가 포함된 회귀모형은 유의하였으며(F=9.67, *p*<.001), 이들 변수들에 의해 난임 관련 삶의 질이 35% 설명되었다(Table 5).

## Discussion

본 연구는 보조생식술을 받는 여성을 대상으로 불확실성과 배우자 지지가 난임 관련 삶의 질에 미치는 영향을 확인하여, 난임 여성의 삶의 질을 향상할 수 있는 간호 중재 개발의 기초자료로 제공하고자 시도되었다. 본 연구의 주요 결과를 바탕으로 다음과 같이 논의하고자 한다.

먼저, 본 연구에서 보조생식술을 받는 난임 여성의 불확실성 정도는 총점 50점에서 평균 28.35점으로 나타나, 같은 도구를 사용한 Kim [22]의 연구 30점과 비슷한 수준이었다. 본 연구가 보조생식술을 받는 난임 여성을 대상으로 하여 2회 이상의 반복 시술로 인한 실패 경험을 가진 여성을 포함하였고(63.9%), Kim [22]의 연구는 체외수정 시술 실패 경험이 있는 여성을 대상으로 하였으므로 대상군의 특성이 비슷한 것에 주목할 필요가 있겠다. 난임 시술의 실패 경험은 재시술 여부와 임신 성공에 대한 불확실성을 높이는 요인이 될 수 있으므로[8] 본 연구의 불확실성 정도와 선행연구[22]가 비슷한 점수인 것으로 생각되며, 난임 시술 실패와 반복 시술은 난임 여성의 불확실성을 증가시킬 수 있으므로, 향후 이러한 요인을 고려하여 대상자의 시술 경험에 따른 차별화된 관리 전략이 필요하다고 본다.

다음으로, 본 연구에서 대상자의 배우자 지지 점수는 평균 86.67점으로 나타나, 같은 도구를 사용한 Park [19]의 연구 82.63점보다는 높았다. 이는 연구대상자의 난임 관련 특성에서 본 연구의 대상자는 보조생식술을 평균 1회 이상 받은 여성을 대상으로 한 반면, Park [19]의 연구에서는 난임을 진단받았지만 체외수정과 같은 보조생식술을 시행하지 않는 여성들을 상당수(37.3%) 포함하고 있어서 본 연구와 대상자 특성에서 차이가 있었기 때문으로 생각된다. 문헌에 따르면 보조생식술은 부부의 스트레스를 증가시키고 관계 변화를 유발하는 원인이 될 수 있는데, 힘들고 어려운 보조생식술 과정을 부부가 함께 극복하면서 오히려 배우자 지지가 강화된다고 설명하기도 하므로[23], 보조생식술을 받고 있는 본 연구대상군의 특성이 일부 반영된 결과라고 생각해 볼 수 있겠다.

본 연구에서 보조생식술을 받는 여성의 난임 관련 삶의 질 평균은 100점 만점에 평균 57.98점으로 나타나, 같은 도구를 사용한 Jung과 Kim [5]의 연구에서 보인 53.8점보다는 높았다. 이러한 차이에 대한 논의는 신중해야 하겠으나, 선행연구와의 차이는 자료수집 장소와 대상자 관리 방식의 차이와 관련될 수 있다고 본다. 즉, 본 연구는 난임 전문병원인 1차 의료기관에서 수행된 반면 Jung과 Kim [5]의 연구는 국내 3차 대학병원의 난임 클리닉에서 수행되었다. 일반적으로 난임 여성이 임산부를 보면 질투와 자존심 상함, 수치감 등으로 위축되어 고독감과 우울함에 빠질 수 있다는 보고를 고려할 때[24], 진료환경에서 임산부를 만나지 않는 난임 전문병원이 난임 여성에게는 좀 더 편안할 수 있으리라고 본다. 또한 보조생식술을 받는 동안 개인이 처한 환경이나 시술 받는 난임 병원의 치료적 환경과 의료인 지지가 난임 전문병원과 3차 의료 기관이 다를 수 있고, 이로 인해 난임 여성이 받는 심리·정서적 반응과 스트레스 정도 및 삶의 질 정도가 다를 수 있다. 그러므로 향후, 병원 치료환경의 차이와 의료인 지지 환경의 차이가 난임 여성의 삶의 질에 미치는 영향을 탐색하는 연구가 필요하다고 본다.

한편, 본 연구에서 보조생식술을 받는 난임 여성의 삶의 질 평균 점수인 57.98점은 같은 도구를 사용한 Kim과 Shin [4]의 연구에서 보인 69.59점보다는 낮았다. 이러한 차이는 대상자의 난임 시술 관련 특성으로 논의할 수 있을 것이다. 즉, 선행연구에서는 체외수정을 하지 않은 대상자의 비율이 각각 75.9%를 차지하였던 것에 비해, 본 연구의 대상자는 보조생식술을 1회 이상 받은 난임 여성으로만 한정하였다. 이는 난임 치료의 마지막 단계인 체외수정 및 배아 이식 과정 자체가 난임 여성에게 심신의 부담감 및 다양한 감정 변화를 일으키고[7], 더욱이 배아 이식 후 임신 확진까지 기다리는 기간은 스트레스가 상당히 높다고 보고되고 있다[8]. 이러한 점에서, 보조생식술 과정에서의 높은 스트레스는 난임 관련 삶의 질 저하에 영향을 줄 수 있으리라고 생각된다. 그러나 본 연구에서 난임 관련 스트레스를 구체적으로 조사하여 선행연구와 비교해 본 것은 아니므로, 해석에 신중해야 할 것이다.

본 연구의 난임 관련 삶의 질 하위 영역을 살펴보면, 관계영역이 평균 62.28점으로 가장 높게 나타났다. 구체적으로, 여러 삶의 질 측정 영역 중 배우자와의 의사 소통 및 애정과 헌신, 성적 관계 만족도를 살펴볼 수 있는 관계 측면의 삶의 질이 상대적으로 높은 것으로 설명되었다. 이러한 결과는 Boivin 등[21]의 연구, Yang과 Yeo [6]의 연구에서 관계영역이 각각 68.7점, 66.7점으로 가장 높게 나타난 것과 일치한 결과이다. 이는 난임 치료과정이 난임 부부가 어려움을 공동으로 견뎌내야 하는 과정이기에 부부 서로간의 생각과 느낌을 공유하고 서로 지원하면서 관계가 더 강화된다는 선행연구[23]의 결과를 지지하는 결과라고 본다.

또한 본 연구의 난임 관련 삶의 질 하위 영역 중 치료환경 영역이 평균 52점으로 가장 낮게 나타났다. 이러한 결과는 같은 도구를 사용하여 미국 등 여러 국가의 난임 진단을 받은 여성과 남성을 대상으로 한 Boivin 등[21]의 연구에서 정서영역이 45.10점으로 가장 낮았던 결과와는 차이가 있었다. 즉, 한국 난임 여성들은 난임 치료환경의 질이 가장 낮다고 응답한 것에 비해, 미국 등의 외국 난임 여성들에게서는 치료환경의 질이 가장 낮지는 않았다. 이러한 면은 아직까지도 한국의 난임 치료환경에 대한 여성들의 만족도가 낮은 것으로 이해할 수 있겠다. 현재 시행되고 있는 난임 부부 지원사업은 2019년 7월부터 난임 시술비 지원에 대한 나이 제한을 없애고 지원 횟수도 늘렸지만[25], 난임으로 인해 생길 수 있는 부정적 감정과 정서들을 없앨 방안은 여전히 부족하고, 일부 기관에서만 필요할 때 받을 수 있다. 또한 난임 여성을 이해하고 그들을 지지하는 역할을 할 수 있는 난임 전문 인력이 부족하여, 난임 여성들은 필요한 정보와 교육을 제대로 받지 못해 답답함과 불확실성을 가진 채[7] 난임 시술을 받을 수밖에 없으며, 우울을 경험하는 경우도 많다[12]. 그러나 이러한 심리적 어려움을 함께 고려한 난임 치료환경의 구축은 미약한 것으로 보인다. 따라서 이러한 어려움을 공감하고 지지하는 역할을 할 수 있는 난임 관련 임상 전문가의 양성이 시급하며, 난임 임상 전문가에 의한 다각도의 관리와 지지가 필요하다고 생각한다.

본 연구에서 회귀분석을 통하여 난임 여성의 삶의 질에 미치는 영향요인을 탐색한 결과, 주요 독립변수인 불확실성은 유의한 영향요인으로 설명되었으며, 불확실성이 높을수록 난임 관련 삶의 질이 낮아지는 것으로 나타났다. 이러한 결과는 난임 여성 대상의 불확실성과 삶의 질 탐색 연구가 드문 실정에서 직접 비교는 어렵지만, 불확실성이 유방암 환자들의 삶의 질을 낮추는 것으로 설명한 연구결과를 지지하는 결과이다[9]. 불확실성이 삶의 질에 미치는 영향은 난임 여성 보다는 다른 질병군에서 설명되고 있는데[9], 본 연구를 통해 난임 여성에서도 불확실성이 삶의 질을 낮추는 주 요인으로 설명된 점이 본 연구의 의의라고 본다. 특히 체외수정에 실패한 난임 여성일수록 더욱 심한 정서적 변화를 경험하게 되므로[7], 난임 치료가 반복됨에 따라 불확실성이 더욱 증가할 수 있다. 따라서 난임 여성의 불확실성을 감소시키기 위해서는 난임 치료의 성공률을 높이기 위한 중재가 기본이 될 것이며, 반복되는 실패를 경험하는 여성을 고위험군으로 관리할 필요가 있다고 본다. 특별히 국외 난임 여성을 대상으로 한 마음 챙김 명상 프로그램은 자기 대처, 부정적 감정 조절, 능동적, 수동적 회피를 개선시킴으로써 임신 성공률을 증가시키고 난임 관련 삶의 질을 향상시키는 것으로 나타났으므로[26], 난임 여성을 대상으로 한 감정 조절 및 대처 향상 프로그램이 불확실성 감소에 도움이 될 것이라고 본다. 또한 난임 여성은 주로 부부 및 가족관계 등의 관계적 측면에 대한 불확실성과, 자신이 현재 하고 있는 치료가 최선이며 잘 진행되고 있는지 등 치료 절차에 대한 불확실성을 크게 느끼는 것을 고려할 때[7,16], 난임 여성뿐 아니라 배우자를 대상으로 하는 부부 의사소통 증진 프로그램과 함께 불임치료 과정에 대한 구체적인 교육과 안내, 지지 등도 중요하다고 본다. 따라서 추후 불확실성 중재 개발 시 이러한 내용을 포함하는 난임 여성과 가족을 위한 불확실성 중재 프로그램의 개발과 적용이 필요하다고 본다.

다음으로, 보조생식술 총 횟수 역시 난임 관련 삶의 질에 큰 영향을 미치는 변수로 설명되었으며, 보조생식술 횟수가 증가할수록 난임 관련 삶의 질이 낮아지는 것으로 나타났다. 이는 Karabulut 등[27]의 연구에서 장기간의 난임 치료가 난임 관련 삶의 질에 부정적인 영향을 준다는 연구결과와 일치하였다. 그리고 Hwang [12]의 연구에서 체외수정 횟수가 증가할수록 우울 정도가 높아진다는 결과와도 관련될 수 있어, 난임 시술 횟수의 증가는 일관성 있게 우울 등의 부정적인 정서를 증가시켜 난임 여성의 삶의 질 저하에 영향을 미칠 수 있다는 것이 재확인되었다. 그러나 보조생식술의 회당 임신율은 30–40%이고[3], 나이가 많을수록 보조생식술의 회당 임신 성공률은 감소한다고 보고되고 있어[15], 보조생식술의 반복 시술은 매우 흔하다. 따라서 여러 차례 반복된 보조생식술 과정에서 약물로 인한 부작용이나 어려움을 최소화하고 우울 등의 부정적 감정을 예방할 수 있도록 돕는 노력이 중요하며, 가능하면 첫 시술의 성공률을 높이기 위한 최선의 노력이 필요할 것이다.

본 연구의 결과 보조생식술을 받는 여성의 주관적 건강 상태가 난임 관련 삶의 질에 영향을 미치는 요인으로 나타났다. 이는 Lee와 Park [11]의 연구에서 난임 여성이 주관적으로 인지하는 건강 상태가 난임 관련 삶의 질에 미치는 유의한 영향이 없었던 결과와는 차이가 있었다. 그러나 난임 여성을 대상으로 한 연구는 아니지만, 국내 젊은 기혼 성인 여성을 대상으로 한 Park [28]의 연구에서 주관적 건강 상태가 건강 관련 삶의 질에 주요한 영향을 미치는 것으로 나타난 연구결과와는 동일하였다. 난임 여성은 반복된 보조생식술로 인해 심적 부담감 및 피로감과 신체적 부작용이 생김에 따라 건강 상태가 떨어지고, 약해지는 악순환의 고리를 가지게 되므로[29] 난임 시술 초기 단계부터 건강한 생활습관 유지와 난임 여성의 건강 상태에 영향을 미칠 수 있는 심리적 스트레스 중재 전략 등을 제공해야 할 필요가 있겠다.

본 연구에서 결혼 기간이 난임 관련 삶의 질에 영향을 미치는 요인으로 나타났으며, 결혼 기간이 증가할수록 난임 관련 삶의 질은 낮았다. 이러한 결과는 결혼 기간에 따라 난임 관련 삶의 질에 유의한 차이가 나타난 선행연구 결과와 일치하였다[12]. 결혼 기간이 길다는 것은 난임 기간이 길다는 측면으로 이해할 수 있을 것이며, 난임 기간이 긴 것은 임신 성공에 대한 불안과 스트레스 증가로 이어질 수 있어[8] 난임 관련 삶의 질이 낮아질 수 있다고 본다. 반면, 첫 보조생식술을 시행하는 데 결혼 기간이 길지 않은 난임 여성은 시술 과정 동안 부부 관계의 결속과 부부 친밀감이 높아져 삶의 질이 높아진다는 것이 선행연구[23]에서 설명되었다. 따라서 부부를 대상으로 하는 간호 중재를 계획할 때 결혼 기간에 대한 고려도 필요할 것이라고 생각되며, 특히 결혼 기간과 난임 기간이 길고 시술 횟수가 많은 난임 여성일수록 부정적 정서를 완화하고, 삶의 질을 향상할 수 있는 중재 방안이 필요하다고 본다.

본 연구에서 난임 검사비용이 난임 관련 삶의 질에 영향을 미치는 요인으로 나타나, 난임 검사비용 부담이 적은 군이 난임 관련 삶의 질이 높았다. 이는 난임 시술비용 부담이 높을수록 난임 관련 삶의 질이 낮아진다는 선행연구[5,10,11]의 결과와 유사하였다. 저출산 대책으로 2019년 7월부터 난임 부부 지원사업은 체외수정 시술(신선배아) 지원 횟수가 2018년 4회에서 최대 7회, 동결배아 5회, 인공수정 5회[25]로 늘어나 시술비에 대한 경제적 부담이 감소할 것으로 기대했으나, 소득과 가족 수별 건강보험료 기준으로 지원 대상자가 한정되어 있어 난임 시술 여성들에게는 여전히 난임 검사 및 치료비용 부담이 남아 있으므로 정부 차원의 지속적인 개선 노력이 요구된다.

본 연구의 결과, 난임 상태에 부담 주는 사람이 있는지가 난임 관련 삶의 질에 영향을 미치는 것으로 나타났으며, 난임에 부담 주는 사람이 있는 군이 없는 군에 비해 난임 관련 삶의 질이 유의하게 낮았다. 이는 Park [19]의 연구에서 난임에 부담 주는 사람이 난임 스트레스에 유의하게 영향을 미치는 요인으로, 난임에 부담을 주는 사람이 있을 때 난임 스트레스가 높아진다고 설명한 맥락과도 관련된다고 본다. 난임 스트레스는 난임 여성의 삶의 질에 주요한 영향을 미치는 요인으로 보고되므로[11], 난임 상황에 부담을 주는 이가 있을 때 난임 관련 스트레스가 증가되고, 나아가 여성의 삶의 질이 감소하는 것으로 볼 수 있겠다. 임신과 출산의 의미를 중요하게 여기는 가족 분위기와 남의 이목을 중시하는 한국인의 성향으로 난임이라는 사실을 주변에 숨기고 주변의 지지 체계를 활용하지 않는 경향 때문에 난임 스트레스를 경험하며, 난임에 부담을 주는 사람이 많다고 느낄 수 있다. 따라서 난임이 부부뿐 아니라 가족이 함께 돕고 해결해야 할 문제라는 사회적 인식 전환을 위한 다각적인 노력이 필요하다[10]. 이를 위해 난임 부부를 대상으로 하는 난임 강의나 프로그램, 자조 모임을 통해 올바른 정보와 인식을 할 수 있는 환경을 조성할 필요가 있다. 그리고, 난임 간호사들은 난임 대상자의 면담과 치료에 참여할 때 이들의 심리·정서적 문제에 대한 민감성을 높이고, 면밀한 사정을 통해 대상자의 문제를 조기에 찾아내어 적절한 중재 전략을 세워야 할 것이다.

본 연구에서 배우자 지지는 불확실성과 유의한 음의 상관관계, 그리고 난임 관련 삶의 질과는 유의한 양의 상관관계가 있음이 확인되었고, 불확실성과 배우자 지지만을 포함한 회귀모형 1에서는 난임 관련 삶의 질의 유의한 영향요인으로 설명되었으나, 단변량에서 난임 관련 삶의 질 관련 변인들로 설명된 일반적 특성들을 포함한 회귀모형 2에서는 유의성이 없었다. 이러한 결과는 유방암 여성을 대상으로 한 선행연구에서 가족 지지가 삶의 질에 유의한 상관관계로 나타났지만, 통제변수를 포함한 회귀분석에서는 유의한 영향을 미치는 요인으로 분석되지 않은 것과 유사한 결과이다[30]. 이는 다양한 측면의 해석이 가능하겠으나, 배우자 지지가 난임 여성의 삶의 질에 영향을 미치는 변수이기는 하지만 난임 여성의 삶의 질에 직접적인 영향을 미치기보다는 불확실성이나 다른 변수를 통하여 삶의 질에 영향을 미치는 간접효과를 지닌 변수라는 것을 시사한다고도 볼 수 있으며, 통제변수로 포함된 다른 변수의 영향이 배우자 지지의 영향에 비해 상대적으로 높았기 때문에 초래된 결과일 수도 있겠다. 따라서, 배우자 지지가 삶의 질에 미치는 직·간접 효과, 혹은 조절 효과 등을 규명하기 위한 후속연구가 필요할 것이라고 본다. 더불어, 배란유도 방법 역시 회귀분석에서는 영향요인으로 나타나지는 않았지만, 단변량 분석에서는 배란유도 방법에 따른 삶의 질에 차이가 있었다. 즉, 자연배란 유도로 보조생식술을 받는 여성이 과배란 유도로 보조생식술을 받는 여성보다 난임 관련 삶의 질 점수가 낮은 것으로 나타났다. 이는 난소 기능 저하로 배란유도 주사에 대한 반응이 저조한 여성이나 나이가 많은 여성에서 주로 자연배란 유도를 이용하여 체외수정 시술을 시행하게 되며[15], 이 경우 단지 하나의 난자만이 배출되고, 난자 배출의 정확한 시기를 예측하기 어렵기 때문에 시술 주기당 성공률도 10% 미만으로 낮기 때문이라고 본다[15]. 따라서 임상에서는 자연배란 유도를 통한 보조생식술을 받는 여성에게 좀 더 관심을 기울여야 할 것이다.

본 연구는 한 도시 소재 1개 기관의 외래 진료 환자를 편의 추출하여 수집한 결과이므로 일반화에는 신중을 기해야 한다. 또한 자가보고 형식에 의한 조사로 연구대상자들이 민감한 질문에 대해서는 솔직한 응답을 하지 않았을 가능성이 있다. 그러나 본 연구는 보조생식술을 받는 난임 여성만을 대상으로 하여, 보조생식술 과정에 있는 난임 여성의 불확실성과 삶의 질 정도에 대한 이해를 도모하고, 이들이 경험하는 불확실성이 난임 관련 삶의 질에 영향을 준다는 점을 확인함으로써, 난임 여성을 대상으로 하는 간호 중재에서 불확실성의 중재가 중요하다는 중재 방향을 제시했다는 점에서 연구의 의의가 있다. 또한, 난임 관련 삶의 질에 영향을 미치는 다양한 영향요인들을 규명하여, 난임 간호 중재 프로그램 적용의 고위험군에 대한 아이디어를 제공하고, 중재 프로그램 개발의 기초자료를 제공한 점에서 실무적 의의가 크다고 본다.

## Conclusion

본 연구는 보조생식술을 받는 여성의 불확실성과 배우자 지지가 난임 관련 삶의 질에 미치는 영향을 파악하고자 시도되었다. 연구결과, 불확실성, 보조생식술 횟수, 주관적 건강 상태, 결혼 기간, 난임 검사비용 부담, 난임에 부담 주는 사람 유무가 난임 관련 삶의 질에 영향을 미치는 것으로 나타났다. 따라서 난임 여성의 난임 관련 삶의 질을 향상시키기 위하여 간호 실무 현장에서 보조생식술 시술 여성의 불확실성 요소를 조기에 사정하여 해결해 줄 수 있는 중재가 필요하며, 이를 위해 질적 연구 등을 통하여 보조생식술을 받는 난임 여성이 느끼는 불확실성에 대한 깊이 있는 탐색 연구가 필요하다고 본다. 또한 본 연구에서 유의한 변수로 설명된 일반적 특성 및 난임 관련 특성을 고려한 대상자별 맞춤형 중재 프로그램을 개발하고 첫 내원 시기부터 난임 치료 단계별로 맞춤형 중재를 제공함으로써, 난임 여성의 삶의 질을 향상시키기 위한 향후 중재 프로그램 개발과 적용이 필요할 것이다.

본 연구의 결과를 토대로 다음과 같이 제언하고자 한다.

첫째, 난임 여성의 불확실성에 대한 보다 깊이 있는 탐색을 위한 질적 연구의 진행을 제언한다.

둘째, 본 연구에서 난임 관련 삶의 질에 미치는 영향요인을 고려한 난임 간호 중재 프로그램 개발과 그 효과를 검증하는 연구를 제언한다.

셋째, 배우자 지지가 삶의 질에 미치는 직·간접 효과를 규명하기 위한 추후의 반복 연구를 제언한다.

넷째, 여러 지역의 다양한 병원에서 반복하여 자료를 수집하여, 연구결과 타당성 확보의 근거를 마련하기 위한 반복 연구를 제언한다.

## Figures and Tables

**Table 1.** Levels of uncertainty, spousal support, and infertility-related quality of life (N=172)

Variable	Categories	Number of items	Mean ± SD	
			Scale level	Item level
Uncertainty	Relational aspect	4	11.91 ± 3.42	2.90 ± 0.86
	Personal aspect	6	16.43 ± 4.84	2.74 ± 0.81
	Total	10	28.35 ± 7.18	2.84 ± 0.72
Spousal support	Support by love, respect, and trust	9	33.36 ± 5.87	3.71 ± 0.65
	Support by communication	2	7.47 ± 1.65	3.73 ± 0.82
	Support by help and care	12	45.85 ± 8.07	3.82 ± 0.67
	Total	23	86.67 ± 14.62	3.77 ± 0.64
Infertility-related quality of life	Emotional domain	6	59.08 ± 17.22	2.36 ± 0.69
	Mind/body domain	6	56.56 ± 18.95	2.26 ± 0.76
	Relational domain	6	62.28 ± 19.66	2.49 ± 0.79
	Social domain	6	58.62 ± 17.26	2.35 ± 0.69
	Environment domain	6	52.74 ± 12.77	2.11 ± 0.51
	Tolerability domain	4	60.00 ± 15.57	2.40 ± 0.62
	Total	34	57.98 ± 12.96	2.32 ± 0.52

**Table 2.** Correlations among uncertainty, spousal support, and infertility-related quality of life (N=172)

Variable	r (*p*)		
	Uncertainty	Spousal support	Infertility-related quality of life
Uncertainty	1		
Spousal support	–.22 (.004)	1	
Infertility-related quality of life	–.28 (< .001)	.23 (< .003)	1

**Table 3.** Differences in infertility-related quality of life by subjects’ characteristics (N=172)

Variable	Categories	n (%)	Mean ± SD	t or F	*p*
Age (year)	< 35	133 (77.3)	56.89 ± 13.10	–2.15	.035
	≥ 35	39 (22.7)	61.67 ± 11.89		
Marriage duration (month)	< 36	54 (31.4)	63.48 ± 10.22	4.32	< .001
	≥ 36	118 (68.6)	55.46 ± 13.33		
Perceived health status	Healthy	155 (90.1)	58.96 ± 12.03	2.28	.035
	Unhealthy	17 (9.9)	49.01 ± 17.55		
Educational level	High school	16 (9.3)	52.67 ± 12.25	1.81	.166
	College	143 (83.1)	58.27 ± 12.96		
	Graduate school	13 (7.6)	61.26 ± 12.74		
Employment	Yes	110 (64.0)	59.22 ± 12.35	1.63	.106
	No	62 (36.0)	55.78 ± 13.80		
Economic status	Low	17 (9.9)	54.07 ± 14.46	0.88	.415
	Medium	126 (73.3)	58.52 ± 12.41		
	High	29 (16.9)	57.91 ± 14.42		
Religion	Protestantism	50 (29.1)	59.81 ± 14.68	0.53	.664
	Catholicism	31 (18.0)	58.06 ± 12.04		
	Buddhism	22 (12.8)	56.65 ± 9.73		
	None	69 (40.1)	57.03 ± 13.04		
Abortion experience	None	107 (62.2)	58.92 ± 12.35	1.17	.312
	One time	40 (23.3)	57.59 ± 13.19		
	Twice or more	25 (14.5)	54.56 ± 14.96		
Person who wants to have a child	Husband	102 (59.3)	57.21 ± 12.71	0.99	.395
	Parents-in-law	25 (14.5)	56.09 ± 14.51		
	Parents	22 (12.8)	61.03 ± 11.47		
	None	23 (13.4)	60.52 ± 13.62		

**Table 4.** Differences in infertility-related quality of life by infertility-related characteristics (N=172)

Variable	Categories	n (%)	Mean ± SD	t or F	*p*
Cause of infertility	Female	58 (33.7)	56.12 ± 14.36	0.70	.553
	Male	10 (5.8)	57.57 ± 15.12		
	Both	21 (12.2)	60.19 ± 11.20		
	Unknown	83 (48.3)	58.76 ± 12.12		
Total number of ART treatments	1^a^	62 (36.0)	63.06 ± 11.33	13.51	< .001
	2–3^b^	74 (43.0)	57.65 ± 11.89		c < a,b^†^
	4 or more^c^	36 (20.9)	49.90 ± 13.73		
Ovulation induction method	COH	124 (72.1)	60.25 ± 11.82	3.85	< .001
	Natural ovulation	48 (27.9)	52.10 ± 14.01		
Infertility treatment period (month)	< 36	139 (80.8)	59.02 ± 11.98	1.84	.073
	≥ 36	33 (19.2)	53.59 ± 15.93		
Financial burden of infertility testing	No	38 (22.1)	62.79 ± 14.41	–2.64	.009
	Yes	134 (77.9)	56.61 ± 12.23		
Demand for infertility counseling	None	4 (2.3)	65.40 ± 16.67	1.78	.152
	Moderate	39 (22.7)	58.94 ± 12.80		
	A great deal	108 (62.8)	58.97 ± 12.27		
	High	21 (12.2)	54.62 ± 14.62		
The presence of a burdensome person	Yes	93 (54.1)	55.01 ± 13.31	–3.36	.001
	No	79 (45.9)	61.48 ± 11.67		

ART: Assisted reproductive technologies; COH: controlled ovarian hyperstimulation.

† Scheffé test.

**Table 5.** Factors affecting infertility-related quality of life (N=172)

Variable	Model 1				Model 2			
	B	β	t	*p*	B	β	t	*p*
(Constant)	57.2		7.54	< .001	78.01		7.82	< .001
Uncertainty	–.44	–.13	–3.27	.001	–.34	–.19	–2.74	.007
Spousal support	0.15	.07	2.31	.022	0.10	.08	1.18	.241
Age					0.34	.01	0.16	.875
Total number of ART treatments					–4.10	–.24	–2.65	.009
Marriage duration					–4.38	–.16	–2.29	.023
Perceived health status (unhealthy)^†^					–9.53	–.22	–3.38	.001
Controlled ovarian hyperstimulation group^‡^					2.64	.09	1.07	.287
Financial burden of infertility testing (yes)^§^					–5.48	–.18	2.76	.007
The presence of a burdensome person (yes)^II^					–3.47	–.13	2.00	.047
	R²=.11, Adjusted R²=.10, F (*p*)=10.15 (<.001)				R²=.35, Adjusted R²=.31, F (*p*)=9.67 (<.001)			

ART: Assisted reproductive technologies. Dummy variable references were ^†^Perceived health status (healthy), ^‡^Natural ovulation group, ^§^Financial burden (no), and ^ǁ^Presence of burdensome person (no).
